# Electrosurgical units – how they work and how to use them safely

**Published:** 2015

**Authors:** Ismael Cordero

**Affiliations:** Biomedical Service Manager: Gradian Health Systems, New York, USA. ismaelcordero@me.com

**Figure:**
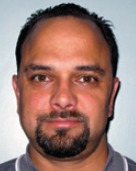
Ismael Cordero

Electrosurgery is used routinely in eye surgery to cut, coagulate, dissect, fulgurate, ablate and shrink tissue. High frequency (100 kilohertz to 5 megahertz), alternating electric current at various voltages (200–10,000 Volts) is passed through tissue to generate heat. An electrosurgical unit (ESU) consists of a generator and a handpiece with one or more electrodes. The device is controlled using a switch on the handpiece or a foot switch.

Electrosurgical generators can produce a variety of electrical waveforms. As these waveforms change, so do the corresponding tissue effects.

In **bipolar electrosurgery** (Figure 1), both the active electrode and return electrode functions are performed at the site of surgery. The two tips of the forceps perform the active and return electrode functions. Only the tissue grasped in the forceps is included in the electrical circuit. Because the return function is performed by one tip of the forceps, no patient return electrode is needed. Bipolar electrosurgery operates regardless of the medium in which it is used, permitting coagulation in a fluid environment – a great advantage when attempting to coagulate in a wet field. As a result, bipolar electrosurgery is often referred to as ‘wet field’ cautery.

In **monopolar electrosurgery** (Figure 2), the active electrode is placed at the surgical site. The patient return electrode (also known as a ‘dispersive pad’ is placed somewhere else on the patient's body. The current passes through the patient as it completes the circuit from the active electrode to the patient return electrode. The function of the patient return electrode is to remove current from the patient safely. A return electrode burn will occur if the heat produced, over time, is not safely dissipated by the size or conductivity of the patient return electrode.

**Figure 1.**
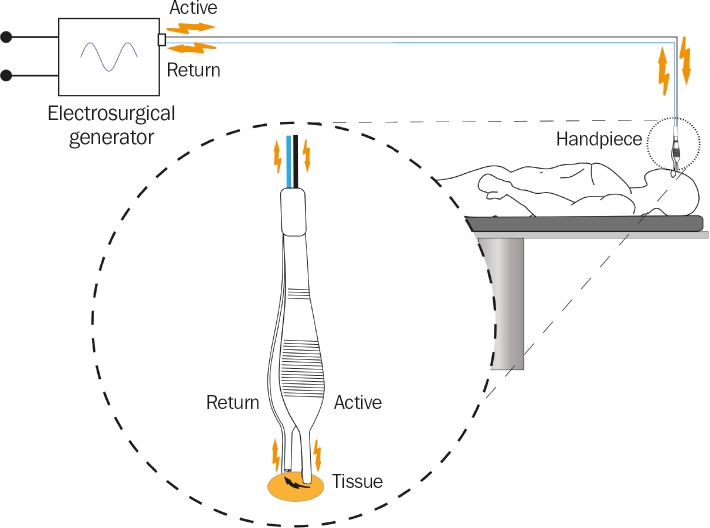
Bipolar electrosurgery

**Figure 2:**
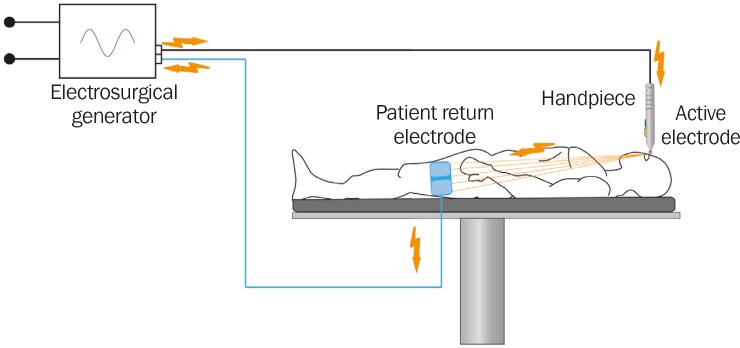


Modern electrosurgical machines have built-in safety features to prevent burns from occurring due to poor contact between the patient and the return electrode when using the monopolar mode.

Often, the term ‘electrocautery’ is incorrectly used to describe electrosurgery. Electrocautery refers to direct current (electrons flowing in one direction) whereas electrosurgery uses alternating current. In electrosurgery, the patient is included in the circuit and current enters the patient's body. During electrocautery, current does not enter the patient's body. Instead, current flows through a heating element, which burns the tissue by direct transfer of heat. Electrocautery or, more precisely, thermocautery units (Figure 3) are usually portable battery powered devices that can be either disposable or reusable.

## Using the ESU safely

ESUs produce very high current that can injure both patient and operator if not properly used and maintained. Many problems have been associated with the use of ESUs, such as burns at the return electrode site and surgical fires. Some of these safety problems can be avoided by taking simple precautions.

### Dos

- The hand piece should always be placed in the nonconductive holster when not in use.
- Always use the lowest possible generator setting that will achieve the desired surgical effect. When higher than necessary voltages are used, the chances of arcing are increased. If the surgeon continues to ask for a higher setting, this could be a signal that the integrity of the skin/dispersive pad interface is compromised.
- Clean the electrode tip frequently. As eschar (dead tissue from burning) builds up on the tip, electrical impedance increases and this can cause arcing, sparking or ignition and flaming of the eschar. When cleaning the electrode, the eschar should be wiped away using a sponge rather than the common scratch pad, because these pads will scratch grooves into the electrode tip, increasing eschar build-up.

**Figure:**
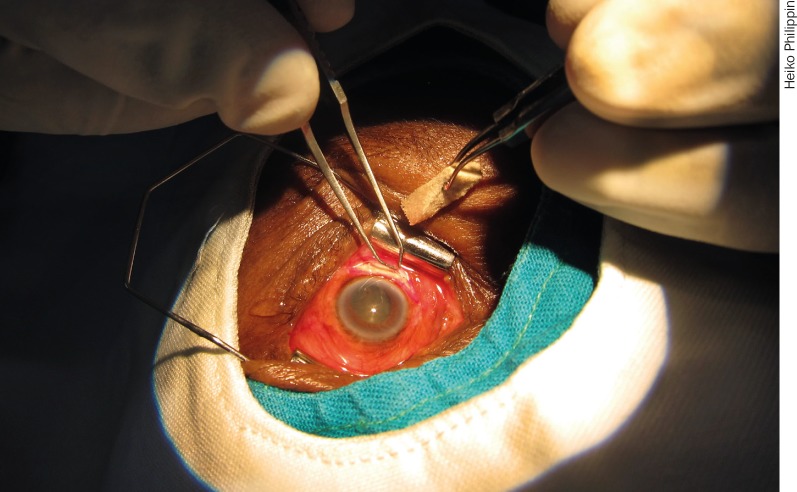
Electrosurgery. TANZANIA

### Don'ts

- ESUs should not be used in the presence of flammable agents or in oxygen-enriched environments.
- Avoid using flammable substances that can be ignited by sparks, such as alcohol and skin degreasers. If you must use alcohol-based skin preps, do not allow them to pool near the dispersive pad; be sure prep solutions are thoroughly dry and fumes have dissipated before ESU activation.
- Rubber catheters or other materials should not be used as a sheath on active electrode tips.
- Cables should never be wrapped around metal instruments, as the current running through them can pass into the metal instrument, causing burns.
- Do not use sharp towel clips or metal instruments to attach cables to drapes. Sharp metal clips can damage electrical cables or provide an unwanted point of contact with the patient's skin. Overlapping electrical wire around a metal clip creates an electrical transformer that can cause a hazard and may ignite drapes.
- Never operate electrosurgical equipment with wet hands or wet gloves. If sterile gloves have holes in them, electrical current can pass through. Be sure that all team members at the surgical field have intact gloves.
- Never operate electrosurgical equipment while standing on a wet surface. Keep the foot pedal dry. Protect it from fluid spillage by covering it with a clear, waterproof cover.

### Monopolar electrosurgery

- Determine whether the patient has any metal implants, including cardiac pacemakers. There is potential for injury if a patient return electrode is placed on the skin over a metal orthopaedic implant.
- For optimum safety, have the patient remove any jewellery to avoid complications from possible current leakage.
- Position and insulate the patient so that she or he is not touching any grounded metal objects.
- Choose a location for the return electrode/dispersive pad that is as close to the operative site as possible, clean and dry, well vascularised, and over a large muscle mass. Avoid bony prominences, adipose tissue, scar tissue, skin over implanted metal prostheses, hairy surfaces, and pressure points. If necessary, shave very hairy skin at the dispersive pad site. Make sure that conductive gel is moist and uniformly spread all over the contact area and that the dispersive pad achieves uniform contact with the patient's skin.
- Position ECG electrodes away from the electrosurgery site and the current pathway through the body.

**Figure 3.**
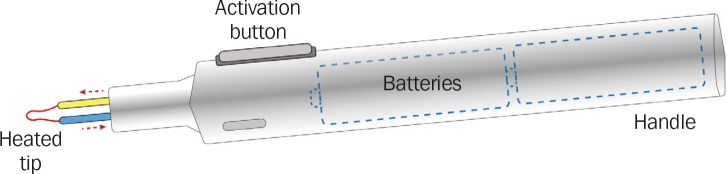
Forceps for electrocautery/thermocautery

